# **Epidemiology of alcohol use disorder in the general population of Togo and Benin: the ALCOTRANS study**

**DOI:** 10.1186/s12889-024-19032-5

**Published:** 2024-06-06

**Authors:** Thibaut Gellé, Aude Paquet, Damega Wenkourama, Murielle Girard, Aurélie Lacroix, Roméo Mèdéssè Togan, Zinsou Selom Degboe, Richard Biaou Boni, Hélène Robin Sacca, Farid Boumediene, Dismand Houinato, Simliwa Kolou Dassa, Didier K. Ekouevi, Pierre- Marie Preux, Philippe Nubukpo

**Affiliations:** 1 Inserm U1094, IRD UMR270, Univ. Limoges, CHU Limoges, EpiMaCT - Epidemiology of Chronic Diseases in Tropical Areas, Institute of Epidemiology and Tropical Neurology, OmegaHealth, Limoges, France; 2 Research and Innovation Unit, Esquirol Hospital Center, Limoges, France; 3 Center for Research in Epidemiology and Population Health, U1018 INSERM, Paris-Saclay University, UVSQ, Villejuif, France; 4 Department of Psychiatry, Faculty of Health Sciences, CHU Kara, University of Kara, Kara, Togo; 5 Faculty of Health Sciences, Department of Public Health, Training and Research Center in Public Health, University of Lomé, Lomé, Togo; 6 African Center for Research in Epidemiology and Public Health (CARESP), Lomé, Togo; 7 Research Action Prevention and Support for Addictions (RAPAA), Lomé, Togo; 8 Faculty of Health Sciences, University Hospital Center of Campus, Clinic of Psychiatry and Medical Psychology of the CHU Campus of Lomé, University of Lomé, Lomé, Togo; 9 Laboratory of Epidemiology of Chronic and Neurological Diseases (LEMACEN), University of Abomey-Calavi, Cotonou, Benin; 10 University Clinic of Neurology of the CNHU-HKM of Cotonou, Cotonou, Benin; 11 National Institute of Health and Medical Research (Inserm), Research Institute for Development (IRD), Bordeaux Population Health Center, University of Bordeaux, UMR 1219, Bordeaux, France; 12 Geriatric Psychiatry and AddictionologyUniversity Hospital Pole of Adult Psychiatry, Esquirol Hospital Center, Limoges, France

**Keywords:** Africa, Togo, Benin, Alcohol use disorder, Epidemiology, Mental health, Public health

## Abstract

**Introduction:**

Access to data concerning mental health, particularly alcohol use disorders (AUD), in sub-Saharan Africa is very limited. This study aimed to estimate AUD prevalence and identify the associated factors in Togo and Benin.

**Methods:**

A cross-sectional study was conducted between April and May 2022, targeting individuals aged 18 years and above in the Yoto commune of Togo and the Lalo commune of Benin. Subjects were recruited using a multi-stage random sampling technique. AUD diagnoses were made using the MINI adapted to DSM-5 criteria. Our study collected sociodemographic information, data on psychiatric comorbidities, stigmatization, and assessed cravings, using a series of scales. The association between AUD and various factors was analyzed using multivariable logistic regression.

**Results:**

In Togo, 55 of the 445 people investigated had AUD (12.4%; [95% CI: 9.5-15.7%]). Among them, 39 (70.9%) had severe AUD and the main associated comorbidities were suicidal risk (36.4%), and major depressive disorder (16.4%). Associated factors with AUD were male gender (aOR: 11.3; [95% CI: 4.8–26.7]), a higher Hamilton Depression Rating Scale (HDRS) score (aOR: 1.2; [95% CI: 1.1–1.3]) and a lower Stigma score measured by the Explanatory Model Interview Catalogue (EMIC) (aOR: 0.9; [95% CI: 0.8–0.9). The stigma scores reflect perceived societal stigma towards individuals with AUD. In Benin, 38 of the 435 people investigated had AUD (8.7%; [95% CI: 6.4–11.7]), and the main associated comorbidities were suicidal risk (18.4%), tobacco use disorder (13.2%) and major depressive episode (16.4%). Associated factors with AUD were male gender (aOR: 6.4; [95% CI: 2.4–17.0]), major depressive disorder (aOR: 21.0; [95% CI: 1.5-289.8]), suicidal risk (aOR: 3.7; [95% CI: 1.2–11.3]), a lower Frontal Assessment Battery (FAB) score (aOR:0.8; [95% CI: 0.8–0.9]) and a lower perceived stigma score (by EMIC )(aOR: 0.9; [95% CI: 0.8–0.9]).

**Conclusion:**

In these communes of Togo and Benin, AUD prevalence is notably high. A deeper understanding of the disease and its local determinants, paired with effective prevention campaigns, could mitigate its impact on both countries.

**Supplementary Information:**

The online version contains supplementary material available at 10.1186/s12889-024-19032-5.

## Introduction

Depressive disorders and alcohol use disorder (AUD) represent significant public health challenges. Together, they account for 50.1% of the causes of loss of healthy life years worldwide [1]. However, data concerning the prevalence of mental disorders vary considerably among countries [2–4]. In sub-Saharan Africa, for instance, the estimated AUD prevalence stood at 6.4% in Benin and 9.5% in Togo as of 2018 [5]. The damage resulting from alcohol consumption has physical, psychological and social dimensions. Neurological issues like epilepsy, neurocognitive disorders, alcoholic neuropathy, and liver diseases are among the severe complications associated with alcohol. A primary concern with alcohol consumption is the development of addiction or severe AUD, marked by loss of control and by the “craving.” This term describes an intense or uncontrollable urge to consume alcohol, even against one’s present wishes [6]. Craving is frequently tied to relapse, which makes it a major focus of addiction management. The co-occurrence of AUD with psychiatric disorders, especially depression, worsens the prognosis of both conditions [7–9]. The overlap of addictive comorbidity and psychiatric diseases intensifies the negative impacts on patients and their families [10] and accentuates the economic pressure associated with these disorders [11, 12]. The sociocultural perceptions that individuals with mental disorders harbor regarding their conditions are crucial for understanding their interactions with society and their internal emotional battles, including issues of self-image and self-esteem. Perceived stigma is a supplementary burden of the disease and profoundly shapes behaviors [13], such as seeking assistance and adhering to treatment. Among the factors linked to AUD, numerous studies have explored sociodemographic parameters such as gender and age. Yet few have examined the link between religion and AUD. While some religions, like Islam, prohibit all alcohol consumption, others incorporate alcohol into rituals, as Christians do with wine. Research has underscored the significance of religion in promoting abstinence [14] and in the stigmatization of individuals with AUD [15–18]. Such stigmatization might push those with AUD to conceal their consumption and resist seeking aid.

Comprehensive data regarding the prevalence, comorbidities, craving, socio-cultural perceptions, and stigmatization tied to alcohol use remain scarce in sub-Saharan Africa. A systematic review focusing on AUD in the general population across sub-Saharan Africa highlighted the existence of 16 studies investigating AUD with validated tools [19]. However, among these studies, none utilized the DSM-5 criteria for AUD diagnosis. The prevalence of AUD varied significantly from one country to another, ranging from 1.0% in Burkina Faso to 31.7% in Kenya, and even within the same country, from 0.1 to 33.2% in Nigeria. The most frequently identified factors associated with AUD included socio-demographic characteristics (notably male gender), low levels of education, and the presence of psychiatric comorbidities. Building upon these findings, our study, ALCOTRANS, aims to contribute to the existing landscape of AUD research in sub-Saharan Africa by specifically utilizing DSM-5 criteria alongside the MINI interview for a comprehensive assessment of AUD and psychiatric comorbidities. This approach addresses the gap identified in the systematic review regarding the diagnostic criteria used but also allows a more precise evaluation of AUD and its complex interplay with psychiatric disorders. Furthermore, ALCOTRANS explores the stigma’s impact on AUD, examining how societal perceptions and self-stigmatization influence the onset and progression of the disorder. By focusing on these novel aspects, ALCOTRANS seeks to provide a more nuanced understanding of AUD within the specific socio-cultural context of Togo and Benin, thereby offering insights that could inform targeted interventions and policy formulations aimed at reducing the burden of AUD in the region.

The current study aims to determine the prevalence of AUD, its associated factors, and comorbidities.

## Methods

### Type and period of study

**Fig. 1:**
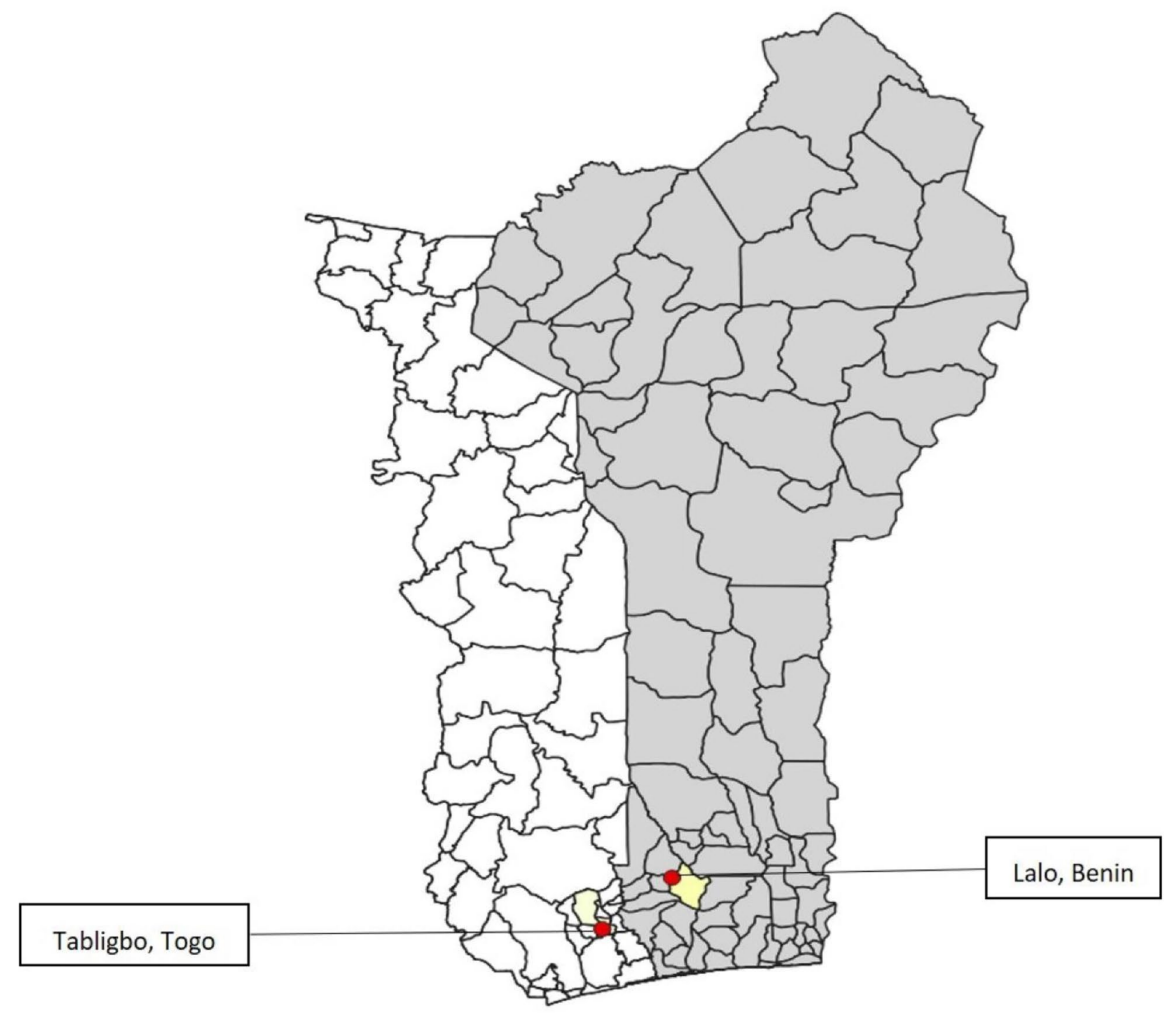
Departments and administrative centers where studies were carried out

A descriptive and analytical cross-sectional study was conducted in Togo and Benin from April 16, 2022, to May 4, 2022. The study targeted the general population within the communes of Yoto (Togo) and Lalo (Benin) (shown in Fig. 1).

### Study area

In Togo, the commune of Yoto is one of the 6 prefectures of Togo’s maritime region, with Tabligbo as its capital. It covers an area of 1,250 km², with a population of 174 851 in 2022 [20]. It is subdivided into three suburban communes, Yoto 1, Yoto 2 and Yoto 3, with Tabligbo, Ahépé and Tokpli as their respective chief towns. In Benin, Lalo is a cosmopolitan suburban commune, representing a junction between several communes in the departments of Couffo, Mono, Zou and Atlantique. The commune of Lalo covers 432 km² with a population of 119,926 inhabitants at the last general population census in 2013 [21]. According to World Bank data, Benin and Togo had literacy rates of 46% and 67% respectively [22]. In Benin and Togo, Christianity is the predominant religion, with 43.5% and 47.8% of the population respectively. Muslims constitute 18.4% of the population in Togo and 27.6% in Benin [23].

By selecting these study areas, we aim to capture the diverse influences on AUD within rural contexts, which are often underrepresented in national data. The choice of Yoto and Lalo for this study is driven by their agricultural significance, demographic characteristics, and the potential to reveal insights into AUD’s prevalence and associated factors within rural African settings.

### Population, sample size and sampling technique

The study involved individuals aged 18 and above who had provided informed consent. Those unable to participate in interviews were excluded. The sample size was calculated according to the Schwartz formula [24] with a prevalence of 10% for AUD, based on the general population data estimated by WHO [5], a precision of 4% in a two-sided situation, an alpha risk of 5% and a design effect of 2. The required number of subjects was 433 per country.

Subjects were recruited using a multi-stage random sampling technique. In the first stage, we randomly selected 6 of the 11 districts in the commune of Lalo, Benin, and 1 of the 3 communes in the district of Yoto, Togo. In the second stage, for both countries, we randomly selected 50% of the villages or neighborhoods in each district. To select the interviewees, investigators went door-to-door in each village. They would start at the village center, spin a pen, and the house indicated by the pen’s tip would be the first to be included. Subsequently, every second house was selected. In each house, every other individual meeting our selection criteria was included. If the required number of participants in a neighborhood was not met, the interviewers returned to the village center to select another direction and repeated the procedure.

### Data collection

The data collection tool was a standardized form digitized in KoboToolbox [25]. Interviewers underwent a 3-day training in addictology and psychiatry led by a specialist to ensure consistent data collection. This training involved role-playing and vernacular explanations of questionnaire concepts. Face-to-face interviews were conducted using smartphones, with each interview averaging about 2 h.

### Variables

The diagnosis of AUD was made using a structured interview for DSM-5 psychiatric diagnoses, inspired by the M.I.N.I. (version 5.0.0, Sheehan DV and Lecrubier Y.) [26] and adapted to DSM-5 criteria by the Addiction team of the SANPSY CNRS/UB UMR 6033 laboratory, for use in an addictological context. Each participant who answered yes to the presence of at least 2 of the 11 criteria over the last 12 months was considered to be suffering from AUD. The severity of the disorder was also assessed using the MINI: mild AUD: 2 to 3 criteria over the past 12 months; moderate AUD: 4 to 5 criteria; severe AUD: 6 or more criteria.

Other variables collected: sociodemographic (age, gender, education, occupation, housing, living environment, family situation, religion) and clinical data (neurological history, treatment and craving status, but only for the AUD population). For psychiatric comorbidities, screening was based on the DSM-5 psychiatric diagnoses, inspired by the M.I.N.I. (version 5.0.0, Sheehan DV and Lecrubier Y). The Hamilton Depression Rating Scale (HDRS) [27] and the Hamilton Anxiety Rating Scale [28] were used to characterize the depressive episode and the anxiety disorder.

Craving in AUD subjects was assessed using a visual analog scale. The subject positions the movable cursor on the ruler, with the left-hand position corresponding to no craving (“No craving at all”) and the right-hand position to maximum craving (“Very high craving”). The result is written on the back of the ruler on a scale from 0 to 10. Zero corresponds to “no craving” and 10 to “very high craving”. We were interested in the average intensity of craving over the past 7 days.

To assess executive functions, we used the Frontal Assessment Battery (FAB) [29]. The total score is a maximum of 18, with higher scores indicating better performance.

The stigma perceived by the study population was explored using the Explanatory Model Interview Catalogue (EMIC) [30]. The EMIC is a semi-structured guide with cross-cultural validity. Its questions can be adapted to suit the objectives of a study. This scale consists of 5 sections aimed at gathering socio-demographic information, details about disease identification or familiarity, as well as perceived causes, stigma, and sought-after assistance related to dementia. We were primarily interested in questions relating to perceived stigma, with a maximum score of 45 points (15 questions scored from 0 to 3). We aimed to quantify the extent of perceived stigma and its impact on individuals with AUD. The higher the scores, the greater the level of perceived stigma.

The internalized stigma of AUD sufferers was explored using the 9-item Stigma Scale [31] validated in French [32]. For each item, the response format is a four-point Likert-type agreement scale ranging from 0 “strongly agree” to 4 “strongly disagree”. The scale measures the extent to which individuals with AUD have internalized societal attitudes towards their condition, with higher scores indicating a greater level of internalized stigma.

### Statistical analysis

Quantitative variables were described by the median and interquartile range according to the normality of the distribution (verified by the Shapiro test). The qualitative variables were presented as numbers and their percentages.

In bivariate analysis, groups were compared using Pearson’s Chi^2 statistical tests or Fisher’s exact test. Associated factors with AUD were initially identified through bivariate analysis. Variables with a significance level < 0.20 were then included in a multivariable model. The reference group for the multivariable analysis is the group without AUD. A stepwise downward multivariable logistic regression was performed, with the Odds Ratio (OR) and their 95% CI reported. We executed 3 multivariable models, selecting the final model based on the smallest Akaike information criterion (AIC). A p-value < 0.05 was considered statistically significant. The Hosmer-Lemeshow test gauged the model’s predictive accuracy. All statistical analyses were performed using SPSS 28.0.0 software (IBM). The only few missing data encountered in both countries are for the stigma scale (*n* = 5 in Togo, *n* = 9 in Benin) and the EVA craving (*n* = 5 in Togo, *n* = 9 in Benin).

## Results

### Socio-demographic and characteristics of participants

**Fig. 2:**
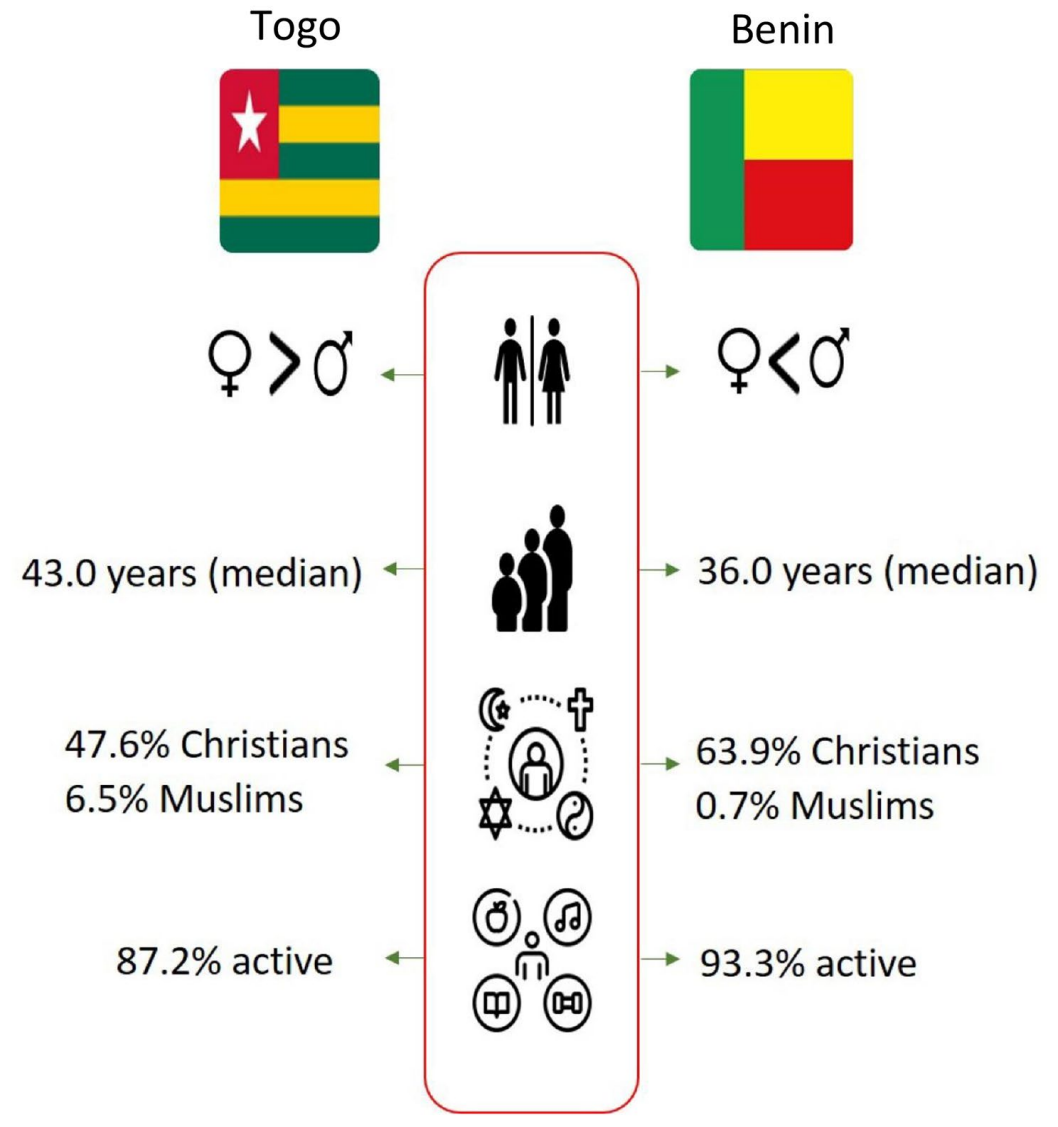
Main characteristics of the samples from both countries

A total of 445 subjects were enrolled in Togo, and 435 in Benin. In Togo, the participation rate in the study was 95.0% and the median age was 43.0 years (interquartile range: 32.5–56.0), with the most prevalent age group being 50.0 years and above, accounting for 37.5% (*n* = 167). In Benin, the participation rate in the study was 97.3% and the median age was 36.0 years (interquartile range: 26.0–48.0), and the predominant age group was 18.0–35.0 years. Descriptions of the study populations in each country are given in Table 1 and specified in Fig. 2.

**Table 1:** Description of participants’ characteristics in Togo and Benin, 2022

Togo					Benin			
	Total (*n* = 445)	Population without AUD (*n* = 390)	Population with AUD (*n* = 55)	*p*	Total (*n* = 435)	Population without AUD (*n* = 397)	Population with AUD (*n* = 38)	*p*
**Age** *n* **(%)**								
[18–35]	126 (28.3)	115 (29.5)	11 (20.0)		189 (43.5)	179 (45.1)	10 (26.3)	
[35–50]	152 (34.2)	130 (33.3)	22 (40.0)	0.300	148 (34.0)	133 (33.5)	15 (39.5)	0.060
50 and over	167 (37.5)	145 (37.2)	22 (40.0)		98 (22.5)	85 (21.4)	13 (34.2)	
**Sex n(%)**								
Male	200 (44.9)	154 (39.5)	46 (83.6)	**< 0.001**	241 (55.4)	210 (52.9)	31 (81.6)	**0.010**
Female	245 (55.1)	236 (60.5)	9 (16.4)		194 (44.6)	187 (47.1)	7 (18.4)	
**Level of education n(%)**								
Not educated	106 (23.8)	98 (25.1)	8 (14.6)		225 (51.7)	203 (51.1)	22 (57.9)	
Primary	178 (40.0)	144 (36.9)	34 (61.8)	**0.002**	127 (29.2)	115 (29.0)	12 (31.6)	0.400
Secondary	161 (36.2)	148 (38.0)	13 (23.6)		83 (19.1)	79 (19.9)	4 (10.5)	
**Profession n(%)**								
Not active	57 (12.8)	54 (13.8)	3 (5.5)	0.080	29 (6.7)	24 (6.0)	5 (13.2)	0.200
Active	388 (87.2)	336 (86.2)	52 (94.5)		406 (93.3)	373 (94.0)	33 (86.8)	
**Housing n(%)**								
Collective	318 (71.4)	279 (71.5)	39 (70.9)	0.400	347 (79.8)	318 (80.1)	29 (76.3)	0.600
Single family	124 (27.9)	109 (28.0)	15 (27.3)		86 (19.8)	77 (19.4)	9 (23.7)	
Single detached	3 (0.7)	2 (0.5)	1 (1.8)		2 (0.5; 0.1–1.5)	2 (0.5)	0 (0.0)	
**Living environment n(%)**								
Rural	260 (58.4)	225 (57.7)	35 (63.6)	0.400	424 (97.4)	386 (97.2)	38 (100)	0.600
Urban	185 (41.6)	165 (42.3)	20 (36.4)		11 (2.5)	11 (2.8)	0 (0.0)	
**Family status n(%)**								
Single	87 (19.5)	75 (19.2)	12 (21.8)		25 (5.7)	21 (5.3)	4 (10.5)	
In a couple	299 (67.2)	262 (67.2)	37 (67.3)	0.800	333 (76.6)	306 (77.1)	27 (71.1)	0.400
With parents	59 (13.3)	53 (13.6)	6 (10.9)		77 (17.7)	70 (17.6)	7 (18.4)	
**Religion n(%)**								
Christian	212 (47.6)	192 (49.2)	20 (36.4)	**0.008**	278 (63.9)	259 (65.2)	19 (50.0)	0.060
Muslim	29 (6.5)	29 (7.4)	0 (0.0)		3 (0.7)	2 (0.5)	1 (2.6)	
Endogenous	193 (43.4)	159 (40.8)	34 (61.8)		150 (34.5)	133 (33.5)	17 (44.8)	
Other	11 (2.5)	10 (2.6)	1 (1.8)		4 (0.9)	3 (0.8)	1 (2.6)	
**Neurological history n(%)**								
No	418 (93.9)	370 (94.9)	48 (87.3)	0.060	432 (99.3)	395 (99.5)	37 (97.4)	0.200
Yes	27 (6.1)	20 (5.1)	7 (12.7)		3 (0.7)	2 (0.5)	1 (2.6)	
**Taking a treatment n(%)**								
None	398 (89.5)	349 (89.5)	49 (89.1)	0.700	395 (90.8)	365 (91.9)	30 (79.0)	**0.040**
Medication	24 (5.4)	21 (5.4)	3 (5.5)		4 (0.9)	3 (0.8)	1 (2.6)	
Traditional	14 (3.1)	13 (3.3)	1 (1.8)		32 (7.4)	26 (6.5)	6 (15.8)	
Both	9 (2.0)	7 (1.8)	2 (3.6)		4 (0.9)	3 (0.8)	1 (2.6)	
**HDRS median (IqR)**	3 (1.0–6.0)	2 [1–5]	6 [4–12]	**< 0,001**	0 (0.0–2.0)	0 (0–2.0)	2.0 (1.0–4.0)	**< 0.001**
**HARS median (IqR)**	2 (0.0–7.0)	1 (0–5)	8 [3–15]	**< 0.001**	0 (0.0–1.0)	0 (0–1.0)	1.0 (0–3.0)	**< 0.001**
**FAB median (IqR)**	13 (9.0–15.0)	13 [10–15]	11 [8–14]	**0.010**	12 (10.0–15.0)	12.0 (10.0–12.0)	9.5 (7.0–12.0)	**< 0.001**
**EMIC median score (IqR)**	27 (23.0–33.0)	28 [24–34]	23 [18–30]	**< 0.001**	27 (24.0–31.0)	27.0 (24.0–31.0)	24.0 (18.0–27.0)	**< 0.001**
**EVA craving median (IqR)**			*7.0 (5.0–8.0)				5.0** (2.0–7.0)	
**Stigma scale median score**			*21.0 (15.0–24.0)				**18.0 (13.0–18.0)	

*= 5 Missing data **= 9 Missing data

Regarding gender distribution, in Togo, women exceeded men (55.1% vs. 44.9%), whereas in Benin, men were more prevalent (55.4% vs. 44.6%). In terms of religious affiliation, 47.6% of the participants identified as Christians, and 6.5% as Muslims in Togo. In Benin, 63.9% of the sample were Christian and 0.7% were Muslim (shown in Table 1; Fig. 2).

### Prevalence of AUD and clinical characteristics

In Togo, suicidal risk concerned 15.5% of studied population [95%: CI 12.4-19.1%], major depressive disorder 5.6% [95%: CI 3.8-8.1%], post-traumatic stress disorder 4.3% [95% CI: 3.8-8.1%], gambling disorder 4.3%; [95% CI: 3.8-8.1%], and tobacco use disorder 3.6%; [95% CI: 2.2-5.6%]. Fifty-five people (12.4%; [95% CI: 9.5-15.7%]) suffered from AUD. Thirty-nine (70.9%) had severe AUD. The main comorbidities associated with AUD were suicidal risk (36.4%), major depressive disorder (16.4%), tobacco use disorder (14.5%) and pathological gambling (14.5%) (shown in Table 2).

In Benin, suicidal risk concerned 5.5% of studied population [95%: CI 9.5-15.7%] and tobacco use disorder 3.4%; [95%: CI 2.0-5.5%]. Thirty-eight people had AUD (8.7%; [95% CI: 6.4-11.7%]). Of the 38 people with AUD, 12 (31.6%) had severe AUD. The main comorbidities associated with AUD were suicidal risk (18.4%), pathological gambling disorder (13.2%) and major depressive disorder (16.4%) (shown in Table 3).

**Table 2:** Prevalence of mental disorders in Yoto, Togo, 2022

	Cases (*n* = 445)	Prevalence (%)	95% CI	Population without AUD *n* = 390 (%)	Population with AUD *n* = 55 (%)	
**Suicidal risk**	69	15.5	12.4–19.1	49 (12.6)	20 (36.4)	
High suicidal risk	8	1.8	0.9–3.4	5 (1.3)	3 (5.5)	
Medium suicidal risk	8	1.8	0.9–3.4	6 (1.5)	2 (3.6)	
Mild suicidal risk	53	11.9	9.1–15.2	38 (9.7)	15 (27.3)	
**Alcohol use disorder**	55	12.4	9.5–15.7	0 (0)	55 (100.0)	
Severity: Mild alcohol use disorder	11	2.5	1.3–4.2	0 (0)	11 (20.0)	
Severity: Moderate alcohol use disorder	5	1.1	0.4–2.4	0 (0)	5 (9.1)	
Severity: Severe alcohol use disorder	39	8.8	6.4–11.7	0 (0)	39 (70.9)	
**Major depressive disorder**	25	5.6	3.8–8.1	16 (4.1)	9 (16.4)	
**Gambling Disorder**	19	4.3	2.7–6.5	11 (2.8)	8 (14.5)	
Severity: Mild Gambling Disorder	11	2.5	1.3–4.2	6 (1.5)	5 (9.1)	
Severity: Moderate gambling disorder	1	0.2	0.0–1.0	1 (0.3)	0 (0)	
Severity: Severe Gambling Disorder	7	1.6	0.7–3.1	4 (1.0)	3 (5.5)	
**Post-traumatic stress disorder**	19	4.3	2.7–6.5	15 (3.8)	4 (7.3)	
**Tobacco use disorder**	16	3.6	2.2–5.6	8 (2.1)	8 (14.5)	
Severity: Mild tobacco use disorder	2	0.4	0.1–1.4	2 (0.5)	0 (0)	
Severity: Moderate tobacco use disorder	0	0	0-0.6	0 (0)	0 (0)	
Severity: Severe tobacco use disorder	14	3.1	1.8–5.1	6 (1.5)	8 (14.5)	
**Agoraphobia**	8	1.8	0.9–3.4	4 (1.0)	4 (7.3)	
**Social anxiety disorder**	7	1.6	0.7–3.1	6 (1.5)	1 (1.8)	
**Dysthymia**	7	1.6	0.7–3.1	5 (1.3)	2 (3.6)	
**Lifetime Antisocial Personality**	5	1.1	0.4–2.4	0 (0)	5 (9.1)	

CI = Confidence Interval

**Table 3:** Prevalence of mental disorders in Lalo, Benin, 2022

	Cases (*n* = 435)	Prevalence (%)	95% CI	Population without AUD *n* = 397 (%)	Population with AUD *n* = 38 (%)
**Alcohol use disorder**	38	8.7	6.4–11.7	0 (0)	38 (100)
Severity: Mild Alcohol Use Disorder	20	4.6	2.9–6.9	0 (0)	20 (52.6)
Severity: Moderate Alcohol use disorder	6	1.4	0.6–2.8	0 (0)	6 (15.8)
Severity: Severe Alcohol use disorder	12	2.8	1.5–4.6	0 (0)	12 (31.6)
**Suicidal risk**	24	5.5	3.7-8.0	17 (4.3)	7 (18.4)
High Suicidal risk	3	0.7	0.2–1.8	1 (0.3)	2 (5.3)
Medium suicidal risk	3	0.7	0.2–1.8	3 (0.8)	0 (0)
Mild suicidal risk	18	4.1	2.6–6.3	13 (3.3)	5 (13.2)
**Tobacco use disorder**	15	3.4	2.0-5.5	10 (2.5)	5 (13.2)
Severity: Mild Tobacco use disorder	10	2.3	1.2-4.0	6 (1.5)	4 (10.5)
Severity: Moderate Tobacco use disorder	2	0.5	0.1–1.5	1 (0.3)	1 (2.6)
Severity: Severe Tobacco use disorder	3	0.7	0.2–1.8	3 (0.8)	0 (0)
**Psychotic syndrome**	5	1.1	0.4–2.5	4 (1.0)	1 (2.6)
**Gambling Disorder**	3	0.7	0.2–1.8	3 (0.8)	0 (0)
Severity: Mild Gambling Disorder	1	0.2	0.0-1.1	1 (0.3)	0 (0)
Severity: Moderate Gambling Disorder	0	0.0	0-0.6	0(0)	0(0)
Severity: Severe gambling disorder	2	0.5	0.1–1.5	2 (0.5)	0 (0)
**Major depressive disorder**	3	0.7	0.2–1.8	1 (0.3)	2 (5.3)

CI = Confidence Interval

In Togo and Benin, AUD sufferers had higher HDRS and HARS scores, but lower FAB and EMIC scores than those without AUD. In Togo, the mean alcohol craving was 7 (IqR: 5–8) and the median score on the stigma scale was 21 (IqR: 15–24). In Benin, the mean alcohol craving was 5 (IqR: 2–7) and the median score on the stigma scale was 18 (IqR: 13–18) (shown in Table 1).

### Factors associated with AUD

In Togo, the factors associated with AUD were male gender (aOR:11.3; [95% CI: 4.8–26.7]), a higher HDRS score (aOR:1.21; [95% CI: 1.1–1.3]) and a lower EMIC score (aOR: 0.9; [95% CI: 0.8–0.9]) (shown in Table 4a).

**Table 4:** Factors Associated with AUD in Yoto (Togo) and Lalo (Benin), 2022: Multivariable Analysis

	β	Adjusted odds ratio	95% CI	*p*
**a: Factors associated with AUD, Yoto (Togo), 2022, multivariable model**				
*Sex*				
Male (vs. Female)	2.4	11.3	4.8-26.7	<0.001
**HDRS (Global score)**	0.2	1.2	1.1-1.3	<0.001
**EMIC (Global score)**	-0.1	0.9	0.8-0.9	<0.001
Hosmer–Lemeshow X² = 12.8, *p* = 0.1				
**b: Factors associated with AUD, Lalo (Benin), 2022, multivariable model**				
*Sex*				
Male (vs. Female)	1.9	6.4	2.4-17.0	<0.001
**Major depressive disorder**				
Yes (vs. no)	3.1	21.0	1.5-289.8	0.023
**Suicide risk**				
Yes (vs. no)	1.3	3.8	1.2-11.3	0.019
**FAB (Global score)**	**-0.2**	**0.8**	**0.7-0.9**	**0.001**
**EMIC (Global score)**	**-0.1**	**0.9**	**0.8-0.9**	**0.005**

Hosmer–Lemeshow X² = 11.9, *p* = 0.2

In Benin, factors associated with AUD were male gender (aOR: 6.4; [95% CI: 2.4–17.0]), major depressive disorder (aOR: 21.0; [95% CI: 1.5-289.8]), suicidal risk (aOR: 3.8; [95% CI: 1.2–11.3]), a lower FAB score (aOR:0.8; [95% CI: 0.7–0.9]) and a lower EMIC score (aOR: 0.9; [95% CI: 0.8–0.9]) (shown in Table 4b).

## Discussion

This study revealed a prevalence of alcohol use disorders (AUD) of 12.4% in the Yoto commune of Togo and 8.7% in the Lalo commune of Benin. Our findings suggest that in Togo, factors associated with AUD include male gender, depressive intensity as measured by the HDRS, and perceived stigma, as assessed by the EMIC. In Benin, the associated factors were male gender, major depressive disorder (diagnosed using the MINI), suicide risk (identified by the MINI), lower FAB scores, and lower EMIC scores. According to the WHO’s 2018 Global Alcohol and Health Report, the AUD prevalence was 3.7% for the WHO African region, 9.5% for Togo, and 6.4% for Benin [5]. These statistics underline the high prevalence rates we observed in the rural areas of both Togo and Benin.

In West Africa, AUD prevalence has been studied and has shown variable results. A previous study in rural Benin for people aged 18 and over reported an alcohol dependence rate of 3.9% [33], using the Composite International Diagnostic Interview (CIDI) with DSM-IV criteria for diagnosis. This variation in prevalence could be due to the study’s location in northern Benin, where 86.6% of subjects were Muslim. The most recent general population census [21] stated that northern Benin has the highest concentration of Muslims. Religious affiliation appears to influence alcohol consumption patterns and reporting behavior. Many studies emphasize religion’s impact on the onset of AUD and maintaining abstinence [15, 34, 35]. There could be reporting biases due to this prohibition, even though AUD cases exist in Muslim countries [36]. Another survey in both rural and urban Togo within people aged 15 to 64 found that 12.2% of men and 9.9% of women reported harmful alcohol consumption, aligning with our findings [37]. However, this research suggests that AUD prevalence in Togo could be higher than in Benin. Potential reasons might include socio-cultural differences and distinct perceptions about alcohol use and its related disorders between the countries. Prior research in the field of neurology, which examined the sociocultural dimensions of epilepsy across Togo, Benin, and France, revealed variations in attitudes towards epilepsy. These variations had tangible effects on healthcare and the experience of social exclusion. These findings underscored the importance of considering sociocultural attitudes when devising strategies for disease management [38]. This could provide insights into the context of addictions. Further research is essential to determine the cause of these prevalence differences. The AUDIT scale remains the primary tool [39–41], followed by the DSM-IV criteria [33, 42, 43]. To our knowledge, few studies in Africa have used DSM-5 criteria to diagnose AUD.

In Togo and Benin, our study found AUD to be more prevalent among males. Similar trends appear in other general population studies across Africa [39–41, 44–48]. This gender disparity in AUD prevalence might result from the treatment inequalities women face due to heightened gender-based stigmatization [49, 50]. Such stigma can lead to further repercussions for women, possibly causing discreet, excessive alcohol consumption, and even fetal alcohol syndrome (FAS) cases, notably prevalent in South Africa [51, 52]. Nevertheless, the substantial stigma faced by women with mental health challenges, such as AUD, might result in underreporting during door-to-door surveys and inhibit their willingness to seek health care. The occurrence of women ‘drinking in secrecy’ is especially widespread in South Africa and poses a considerable obstacle in accessing treatment [52]. Additional studies in African nations, including Togo and Benin, are essential to grasp the full scope of this issue comprehensively. In Togo, AUD was associated with a higher HDRS (Hamilton Depression Rating Scale) score. Meanwhile, in Benin, AUD correlated with major depressive disorder. This comorbidity with depression mirrors findings from other sub-Saharan Africa studies, especially in Ethiopia [39, 40]. The HDRS measures depression intensity. Even if the overall HDRS score for AUD subjects doesn’t indicate depression (a total score < 21), a one-point increase relates to AUD. To elucidate the link between depressive disorders and Alcohol Use Disorders (AUD), various hypotheses have been explored. One theory suggests that individuals with depressive disorders are at an increased risk of encountering AUD. On the flip side, it is also hypothesized that experiencing AUD may heighten the vulnerability to depressive disorders. Another theory proposes that depressive disorders and AUD might stem from overlapping pathophysiological mechanisms or shared risk factors. The interaction between these conditions could also be influenced by alcohol’s capacity to induce depressive effects, potentially manifesting even in the absence of a diagnosed depressive episode [53]. A significant finding from our study is the pronounced stigma towards individuals with AUD in both countries. Numerous studies have underscored this stigma [54–57], which is often internalized. While sociocultural perceptions and stigmatization have a profound impact on patients, influencing their care and treatment-seeking behavior [17, 57–59], our findings introduce a nuanced perspective. Specifically, individuals with AUD in Togo and Benin exhibited lower EMIC scores than those without AUD, indicating a potentially lower perception of perceived stigma among those diagnosed with AUD. This can reflect some ambivalence, and a lower motivational stage to quit alcohol according to Prochaska and Di Clemente [60] or a self-esteem protection strategy. This underlines a variation in how individuals with AUD perceive, experience, or acknowledge stigma, underscoring the complexity of stigma and its implications for mental health and substance use disorders. There are methods to reduce AUD stigma [61–64]. Educational campaigns and anti-stigma initiatives, aiming to increase public understanding of the disorder, have proven successful in reducing mental health stigma [62, 63]. Enhanced awareness might encourage more AUD subjects to seek help [62]. In Benin, our study shows that AUD adversely impacts cognitive function and increases suicide risk. Substance use disorders, including alcohol, associate with cognitive deficits [65] and a higher suicide risk [66]. Existing evidence supports initiatives to reduce alcohol consumption and raise awareness as preventative measures against suicidal tendencies [67, 68].

Following this study, we shared our findings with academic, political, and medical authorities in both Togo and Benin. All stakeholders thoroughly discussed the results. Topics of conversation covered a range of issues, including current legislation in Benin aimed at combating addictions, such as the ban on selling tobacco products within 500 m of schools. Additionally, the possibility of implementing similar regulations related to alcohol was examined. We agreed with authorities to initiate preventive measures, informing the public about the risks of counterfeit spirits from local distilleries. Raising alcohol taxes might help reduce alcohol consumption prevalence. The discussions also emphasized training community health workers to address suicidal thoughts and risks. Most sub-Saharan countries have not yet implemented all the WHO recommendations to combat alcohol harm [69]. In light of the high prevalence of AUD identified in our study, we underscored the critical need for expanding treatment options and actively encouraging treatment-seeking behaviors among individuals affected by AUD.

This study is the first to provide data from rural areas in both Togo and Benin, forming a basis for comparisons with other rural regions, which remain significant in both countries. Yet, despite their close geography, not all areas showed consistent patterns. Additional research should be conducted across various regions of both countries to enhance the national-level comprehension of this medical condition.

Our study has some limitations. Its cross-sectional design prevents establishing causality between factors and alcohol consumption. Additionally, the wide confidence intervals observed in some of our analyses suggest caution in interpreting these results, as they may indicate variability in the data or relatively small sample sizes for specific subgroups. Though French is the official language in Togo and Benin, with literacy rates of 46% and 67% respectively, both countries have many local languages. Though we used French scales, interviewers had to translate questions when participants couldn’t answer in French, introducing potential translation biases. This might partly explain the higher suicidal risk rates observed in both countries. Overestimation of suicidal risk might arise from linguistic expressions of emotional distress, which doesn’t necessarily reflect genuine intentions. The data collection method, conducted in participants’ homes, is another aspect to consider, especially given the influence of religious beliefs on the perception and reporting of alcohol use in some segments of the population studied. This context could have influenced participants’ willingness to disclose sensitive information, including their alcohol consumption habits. However, it is important to note that interviewers were trained to conduct interviews, thus mitigating potential biases, such as underreporting of alcohol consumption, in data collection.

One of the key strengths of this study is the application of the MINI diagnostic criteria, marking it, to our knowledge, as a groundbreaking investigation into AUD in West Africa. Our multi-stage random sampling allowed us to compare AUD features between the two nations, achieving the necessary sample size. We used validated data collection tools, and the researchers underwent proper training in their application.

## Conclusion

This research reveals high AUD prevalence in Togo and Benin, associating with male gender, perceived stigma, and depression in both nations. In Benin, AUD also relates to cognitive impairment and suicidal risk. Future interventions should prioritize prevention and education campaigns addressing substance use disorders. At the same time, training for primary care providers and other healthcare professionals is crucial. Further AUD research in sub-Saharan Africa, targeting the general population and studying the impact of stigma and sociocultural perceptions, will enhance our understanding of these intricate issues. Adopting the standardized DSM-5 diagnostic criteria could improve result comparability, fostering a deeper global understanding of AUD.

### Electronic supplementary material

Below is the link to the electronic supplementary material.

Supplementary Material 1

## Data Availability

The data that support the findings of this study are not publicly available due to privacy or ethical restrictions. The data that support the findings of this study are available on request from the corresponding author.

## Abbreviations

AIC: Akaike Information Criterion
AUD: Alcohol Use Disorders
aOR: Adjusted Odds Ratio
CI: Confidence Interval
DSM: 5:Diagnostic and Statistical Manual of Mental Disorders, Fifth Edition
EMIC: Explanatory Model Interview Catalogue
FAB: Frontal Assessment Battery
HDRS: Hamilton Depression Rating Scale
HARS: Hamilton Anxiety Rating Scale
IqR: Interquartile Range
M.I.N.I.: Mini International Neuropsychiatric Interview
OR: Odds Ratio
SANPSY: Service d’Addictologie et de Neuropsychiatrie Psychologique et Sexologique
SPSS: Statistical Package for the Social Sciences
