# Supplementary material for: Epidemiology of alcohol use disorder in the general population of Togo and Benin: the ALCOTRANS study
Source: BMC Public Health. 2024 Jun 6;24:1527. doi: 10.1186/s12889-024-19032-5 (PMC11157932; doi:10.1186/s12889-024-19032-5)
Supplement: Supplementary file 1 — Supplementary Material 1 [file 12889_2024_19032_MOESM1_ESM.pdf]

Disclaimer: These scales have not been used in English.

Clinical Data

Visual Analog Scale for Craving **Date Taken:**

Score I___I___I

HARS : *Hamilton Anxiety Rating Scale* **Date Taken:**

Total Score I___I___I

Psychic Anxiety Score: I___I___I

Somatic Anxiety Score: I___I___I

HDRS : *Hamilton Depression Rating Scale* **Date Taken:**

Score I___I___I

BREF: *Echelle Rapide d'efficience frontal* **Date Taken:**

Similarities: I___I
Lexical Evocation: I___I
Prehensile Behavior: I___I
Motor Sequences: I___I
Contradictory Instructions: I___I
Go – No Go: I___I

EMIC : *Explanatory Model Interview Catalogue* **Date Taken:**

Score for perceived social stigma evaluation: I___I___I

ISMIS : *Stigma Scale* **Date Taken:**

Score: I___I___I
Discrimination (questions 5, 6, and 7) =
Transparency (questions 2, 4, and 9) =
Positive Aspects (questions 1, 3, and 8) =

Structured Interview for DSM Psychiatric Diagnostics

for

DSM-5

This questionnaire is inspired by the M.I.N.I. version 5.0.0 (Sheehan DV and Lecrubier Y.) and adapted to DSM-5 criteria by the Addiction Team at the SANPSY CNRS USR 3413, which granted usage authorization to the INSERM 1094 NET team and its collaborators. This document may not be reproduced in whole or in part or transmitted in any form including photocopies or stored on a computer system without authorization.

DISCLAIMER

The purpose of this tool is to assist in the evaluation and screening of patients with greater efficiency and accuracy. Before any exploitation, the data collected and processed by this program must be reviewed and interpreted by a qualified clinician.This program is not designed to replace a complete medical and psychiatric evaluation by a competent, licensed psychiatrist. It is only intended as a tool to facilitate the precise collection and proper treatment of data on symptoms identified by trained personnel. This is not a diagnostic test

Craving Evaluation Scale

Absent

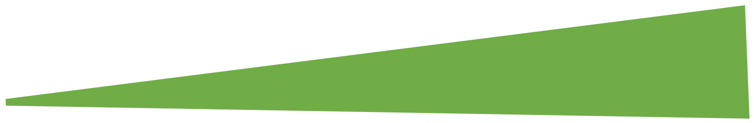

Extremely Important

0	1	2	3	4	5	6	7	8	9	10

**HARS Hamilton Anxiety Rating Scale**	Date :

scoring : 0=none 1=mild 2=moderate 3=severe 4=very severe

		**0**	**1**	**2**	**3**	**4**
1	**ANXIOUS MOOD**<br>Worry - Expecting the worst - Apprehension (anticipation with fear) - Irritability	☐	☐	☐	☐	☐
2	**TENSION**<br>Sensations of tension - Fatigability - Inability to relax - Startle responses - Easy crying - Trembling - Sensation of being unable to remain still	☐	☐	☐	☐	☐
3	**FEARS**<br>Of the dark - Of strangers - Of being left alone - Of large animals, etc. - Of traffic - Of crowds	☐	☐	☐	☐	☐
4	**INSOMNIA**<br>Difficulty falling asleep - Interrupted sleep - Unrefreshing sleep with tiredness upon waking - Distressing dreams - Nightmares - Night terrors	☐	☐	☐	☐	☐
5	**COGNITIVE FUNCTIONS**<br>Difficulty concentrating - Poor memory	☐	☐	☐	☐	☐
6	**DEPRESSIVE MOOD**<br>Loss of interest - No longer enjoys hobbies - Depression - Morning insomnia - Mood swings throughout the day	☐	☐	☐	☐	☐
7	**GENERAL SOMATIC SYMPTOMS (MUSCULAR)**<br>Pains and aches in muscles - Muscular stiffness - Muscular twitches - Clonic jerks - Teeth grinding - Unsteady voice	☐	☐	☐	☐	☐
8	**GENERAL SOMATIC SYMPTOMS (SENSORY)**<br>Ringing in ears - Blurred vision - Hot or cold flashes - Sensations of weakness - Tingling sensations	☐	☐	☐	☐	☐
9	**CARDIOVASCULAR SYMPTOMS**<br>Tachycardia - Palpitations - Chest pain - Beating of vessels - Syncopal sensations - Extrasystoles	☐	☐	☐	☐	☐
10	**RESPIRATORY SYMPTOMS**<br>Weight on the chest or sensation of constriction - Sensation of suffocation - Sighs - Dyspnea	☐	☐	☐	☐	☐
11	**GASTROINTESTINAL SYMPTOMS**<br>Difficulty swallowing - Belching - Dyspepsia - Pain before/after eating - Burning sensations - Bloating - Heartburn - Nausea - Vomiting - Hollow stomach feeling - Abdominal colic - Borborygmi - Diarrhea - Weight loss - Constipation	☐	☐	☐	☐	☐
12	**GENITOURINARY SYMPTOMS**<br>Amenorrhea - Menorrhagia - Onset of frigidity - Frequent urination - Urinary urgency - Premature ejaculation - Absence of erection - Impotence	☐	☐	☐	☐	☐
13	**SYMPTOMS OF THE AUTONOMIC NERVOUS SYSTEM**<br>Dry mouth - Flushes - Pallor - Tendency to sweat - Dizziness - Tension headache - Goosebumps					
14	**BEHAVIOR DURING THE INTERVIEW (GENERAL)**<br>(GENERAL) : Tense, uneasy - Nervous hand movements - Fidgeting with fingers - Clenching fists - Tics - Clutching handkerchief - Back-and-forth instability - Hand tremors - Furrowed brow - Tense facial expression - Increased muscle tone - Gasping breath - Facial pallor<br>(PHYSIOLOGICAL) : Swallowing saliva - Belching - Resting tachycardia - Respiratory rate over 20/min - Lively tendon reflexes - Tremor - Pupillary dilation - Exophthalmos - Sweating - Eyelid fluttering	☐	☐	☐	☐	☐

HDRS Hamilton Depresseion Rating Scale

1 DEPRESSED MOOD *(sadness, hopeless, helpless, worthless)*
0 |__| Absent.
1 |__| These feeling states indicated only on questioning.
2 |__| These feeling states spontaneously reported verbally.
3 |__| Communicates feeling states non-verbally, i.e. through facial expression, posture, voice and tendency to weep.
4 |__| Patient reports virtually only these feeling states in his/her spontaneous verbal and non-verbal communication.

3 SUICIDE
0 |__| Absent.
1 |__| Feels life is not worth living.
2 |__| Wishes he/she were dead or any thoughts of possible death to self.
3 |__| Ideas or gestures of suicide.
4 |__| Attempts at suicide (any serious attempt rate 4).

4 INSOMNIA: EARLY IN THE NIGHT
0 |__| No difficulty falling asleep.
1 |__| Complains of occasional difficulty falling asleep, i.e. more than ½ hour.
2 |__| Complains of nightly difficulty falling asleep.

5 INSOMNIA: MIDDLE OF THE NIGHT
0 |__| No difficulty.
1 |__| Patient complains of being restless and disturbed during the night.
2 |__| Waking during the night – any getting out of bed rates 2 (except for purposes of voiding).

6 INSOMNIA: EARLY HOURS OF THE MORNING
0 |__| No difficulty.
1 |__| Waking in early hours of the morning but goes back to sleep.
2 |__| Unable to fall asleep again if he/she gets out of bed.

7 WORK AND ACTIVITIES
0 |__| No difficulty.
1 |__| Thoughts and feelings of incapacity, fatigue or weakness related to activities, work or hobbies.
2 |__| Loss of interest in activity, hobbies or work – either directly reported by the patient or indirect in listlessness, indecision and vacillation (feels he/she has to push self to work or activities).
3 |__| Decrease in actual time spent in activities or decrease in productivity. Rate 3 if the patient does not spend at least three hours a day in activities (job or hobbies) excluding routine chores.
4 |__| Stopped working because of present illness. Rate 4 if patient engages in no activities except routine chores, or if patient fails to perform routine chores unassisted.

8 RETARDATION (slowness of thought and speech, impaired ability to concentrate, decreased motor activity)
0 |__| Normal speech and thought.
1 |__| Slight retardation during the interview.
2 |__| Obvious retardation during the interview.
3 |__| Interview difficult.
4 |__| Complete stupor.

9 AGITATION
0 |__| None.
1 |__| Fidgetiness.
2 |__| Playing with hands, hair, etc.
3 |__| Moving about, can't sit still.
4 |__| Hand wringing, nail biting, hair-pulling, biting of lips.

10 ANXIETY PSYCHIC
0 |__| No difficulty.
1 |__| Subjective tension and irritability.
2 |__| Worrying about minor matters.
3 |__| Apprehensive attitude apparent in face or speech.
4 |__| Fears expressed without questioning.

2 FEELINGS OF GUILT
0 |__| Absent.
1 |__| Self reproach, feels he/she has let people down.
2 |__| Ideas of guilt or rumination over past errors or sinful deeds.
3 |__| Present illness is a punishment. Delusions of guilt.
4 |__| Hears accusatory or denunciatory voices and/or experiences threatening visual hallucinations.

11 ANXIETY SOMATIC (physiological concomitants of anxiety) such as:
gastro-intestinal – dry mouth, wind, indigestion, diarrhea, cramps, belching
cardio-vascular – palpitations, headaches
respiratory – hyperventilation, sighing
urinary frequency
sweating
0 |__| Absent.
1 |__| Mild.
2 |__| Moderate.
3 |__| Severe.
4 |__| Incapacitating.

12 SOMATIC SYMPTOMS GASTRO-INTESTINAL
0 |__| None.
1 |__| Loss of appetite but eating without staff encouragement. Heavy feelings in abdomen.
2 |__| Difficulty eating without staff urging. Requests or requires laxatives or medication for bowels or medication for gastro-intestinal symptoms.

13 GENERAL SOMATIC SYMPTOMS
0 |__| None.
1 |__| Heaviness in limbs, back or head. Backaches, headaches, muscle aches. Loss of energy and fatigability.
2 |__| Any clear-cut symptom rates 2.

14 GENITAL SYMPTOMS (symptoms such as loss of libido, menstrual disturbances)
0 |__| Absent.
1 |__| Mild.
2 |__| Severe.

15 HYPOCHONDRIASIS
0 |__| Not present.
1 |__| Self-absorption (bodily).
2 |__| Preoccupation with health.
3 |__| Frequent complaints, requests for help, etc.
4 |__| Hypochondriacal delusions.

**a) According to the patient:**	**b) According to weekly measurements:**
0 |__| No weight loss.	0 |__| Less than 1 lb weight loss in week.
1 |__| Probable weight loss associated with present illness.	1 |__| Greater than 1 lb weight loss in week.
2 |__| Definite (according to patient) weight loss.	2 |__| Greater than 2 lb weight loss in week.
3 |__| Not assessed.	3 |__| Not assessed.

17 INSIGHT
0 |__| Acknowledges being depressed and ill.
1 |__| Acknowledges illness but attributes cause to bad food, climate, overwork, virus, need for rest, etc.
2 |__| Denies being ill at all.

Total score: |__|__|

Frontal Assessment Battery *(Dubois, B., Slachevsky, A., Litvan, I., & Pillon, B)*

1 Similitudes (élaboration conceptuelle)

How are these similar:

▶ A banana and an orange ☐ 1

Help the patient in case of total failure: "they are not similar"
or partial: "they both have a peel",
by saying: "an orange and a banana are both..."

Do not help the patient for the following two items

▶ A table and a chair? ☐ 1

▶ A tulip, a rose, and a daisy? ☐ 1

SCORE ____ **/3**

Scoring: Only categorical responses (fruits, furniture, flowers) are considered correct,

3 correct answers = 3
2 correct answers = 2
1 correct answers = 1
no correct answers = 0

2 Lexical Evocation (mental flexibility)

▶ **Name as many different words as possible, for example animals, plants, objects, but not first names or proper names, starting with the letter "S".**

▶ If the patient does not give any response during the first 5 seconds, tell them: "for example, snake". If the patient pauses for more than 10 seconds, stimulate them after each pause by saying "any word starting with the letter S".

Scoring
The duration for this task is 60 seconds; repetitions of words, variations on the same word (e.g., whistle, whistling), and names are not counted as correct responses.

More than 10 words = 3 ☐ 3
From 6 to 10 words = 2 ☐ 2
From 3 to 5 words = 1 ☐ 1
Less than 3 words = 0 ☐ 0

SCORE ____ **/3**

3 Grasping Behavior (Environmental Autonomy)

▶ The examiner is seated opposite the patient whose hands rest on his knees, palms open upwards.

▶ The examiner gently approaches the hands and touches those of the patient, to see if he will grasp spontaneously.

▶ If the patient takes them, ask: "now, do not take my hands anymore."

Scoring

The patient does not take the hands of the examiner = 3 ☐ 3
The patient hesitates or asks what to do = 2 ☐ 2
The patient takes the hands without hesitation = 1 ☐ 1
The patient takes the hands of the examiner, after being asked not to do so = 0 ☐ 0

SCORE ____ **/3**

4 Motor Sequences (Programming)

▶ **"Watch carefully what I do."**
The examiner seated opposite the patient performs alone three times with his left hand the Luria sequence "slice-fist-palm."

▶ "Now, you will perform this sequence with your right hand, first at the same time as me, then alone."

▶ The examiner then performs the sequence three times with his left hand at the same time as the patient, and then tells him: "continue."

Scoring		
	The patient performs alone 6 consecutive correct sequences = 3	☐ 3
	The patient performs alone at least 3 consecutive correct sequences = 2	☐ 2
	The patient performs alone but executes 3 consecutive correct sequences at the same time as the examiner = 1	☐ 1
	The patient cannot perform 3 consecutive correct sequences, even with the examiner = 0	☐ 0
	**SCORE**	**/3**

5 Conflicting Instructions (Sensitivity to Interference)

▶ "When I tap once, you must tap twice."
To ensure that the patient has understood the instruction, the examiner has him perform a sequence of three trials: 1-1-1

▶ "When I tap twice, you must tap once."
To ensure that the patient has understood the instruction, the examiner has him perform a sequence of three trials: 2-2-2. The proposed sequence is as follows: 1-1-2-1-2-2-2-1-1-2

Scoring		
	No errors = 3	☐ 3
	1 or 2 errors = 2	☐ 2
	More than 2 errors = 1	☐ 1
	The patient taps the same number of times as the examiner at least 4 consecutive times = 0	☐ 0
	**SCORE**	**/3**

6 Go – No Go (Inhibitory Control)

▶ "When I tap once, you must tap once."
To ensure that the patient has understood the instruction, the examiner has him perform a sequence of three trials: 1-1-1

▶ "When I tap twice, you must not tap."
To ensure that the patient has understood the instruction, the examiner has him perform a sequence of three trials: 2-2-2. The proposed sequence is as follows: 1-1-2-1-2-2-2-1-1-2

Scoring		
	No errors = 3	☐ 3
	1 or 2 errors = 2	☐ 2
	More than 2 errors = 1	☐ 1
	The patient taps the same number of times as the examiner at least 4 consecutive times = 0	☐ 0
	**SCORE**	**/3**

**TOTAL**		**/18**

Assessment of Social Representations of Alcohol Use Disorders

Adapted EMIC Questionnaire (Explanatory Model Interview Catalogue)
(mark the corresponding box)

		yes		*no*
1	Have you suffered from an alcohol use disorder?	☐		☐
2	Has one of your relatives suffered or is currently suffering from an alcohol use disorder?	☐		☐

		yes	*maybe both (Y/N)*	*no*	*don't know*
3	Is a person suffering from an alcohol use disorder respected as much as any other person in the community?	☐	☐	☐	☐
4	Could being in contact with a person suffering from an alcohol use disorder have negative effects on oneself?	☐	☐	☐	☐
5	Do you think it is possible to have normal contact with a person suffering from an alcohol use disorder?	☐	☐	☐	☐
6	Is it acceptable to enter the house of a person suffering from an alcohol use disorder?	☐	☐	☐	☐
7	Are people suffering from an alcohol use disorder as well appreciated as others in their surroundings?	☐	☐	☐	☐
8	Are the children of people suffering from an alcohol use disorder respected as much as anyone else in the community?	☐	☐	☐	☐
9	If you were married to someone suffering from an alcohol use disorder, would you stay with them? Would you support them during their illness?	☐	☐	☐	☐
10	If you were married to someone suffering from an alcohol use disorder, would you refuse to have sexual relations with them because of this problem?	☐	☐	☐	☐
11	If you were married to someone suffering from an alcohol use disorder, do you think alcoholism would cause you marital problems?	☐	☐	☐	☐
12	Would you agree to marry or allow one of your children to marry a family member of a person suffering from an alcohol use disorder?	☐	☐	☐	☐
13	Would you ask a person suffering from an alcohol use disorder to stay away from other community members (neighborhood) or people at work?	☐	☐	☐	☐
14	Do you think a person suffering from an alcohol use disorder would isolate themselves?	☐	☐	☐	☐
15	Do you think people suffering from an alcohol use disorder have other health problems because of alcohol consumption?	☐	☐	☐	☐
16	If a family member suffered from an alcohol use disorder, would you provide support?	☐	☐	☐	☐
17	If your child's spouse suffered from an alcohol use disorder, would you provide support?	☐	☐	☐	☐

9-Item Stigma Scale (Morandi et al., 2013)

	Strongly agree	Agree	Neither agree nor disagree	Disagree	Strongly disagree
Having had a mental health problem has made me more understanding.					
I am afraid to tell people that I am receiving psychological treatment.					
My mental health issues have made me more tolerant of other people.					
I am afraid of people's reactions if they find out about my mental health issues.					
I am upset by the way people have reacted to my mental health issues.					
People have avoided me because of my mental health issues					
People have insulted me because of my mental health issues.					
Having had mental health issues has made me stronger.					
I find it difficult to tell people that I have mental health issues.					

Information about the Stigma Scale

Scoring: 4="Strongly agree", 3="Agree", 2="Neither agree nor disagree", 1="Disagree", and 0="Strongly disagree"
For questions 1, 3, and 8, scoring is reversed (0="Strongly agree", 4="Strongly disagree")
